# Closing the loop in primate prefrontal cortex: inter-laminar processing

**DOI:** 10.3389/fncir.2012.00088

**Published:** 2012-11-22

**Authors:** Ioan Opris, Joshua L. Fuqua, Peter F. Huettl, Greg A. Gerhardt, Theodore W. Berger, Robert E. Hampson, Sam A. Deadwyler

**Affiliations:** ^1^Department of Physiology and Pharmacology, Wake Forest University School of MedicineWinston-Salem, NC, USA; ^2^Department of Anatomy and Neurobiology, University of KentuckyLexington, KY, USA; ^3^Department of Biomedical Engineering, University of Southern CaliforniaLos Angeles, CA, USA

**Keywords:** prefrontal cortex, inter-laminar correlated firing, nonhuman primates, columnar correlates of target selection, columnar correlates of task difficulty, spatial vs. object tuning

## Abstract

Prefrontal cortical (PFC) activity in the primate brain emerging from minicolumnar microcircuits plays a critical role in cognitive processes dealing with executive control of behavior. However, the specific operations of columnar laminar processing in prefrontal cortex (PFC) are not completely understood. Here we show via implementation of unique microanatomical recording and stimulating arrays, that minicolumns in PFC are involved in the executive control of behavior in rhesus macaque nonhuman primates (NHPs) performing a delayed-match-to-sample (DMS) task. PFC neurons demonstrate functional interactions between pairs of putative pyramidal cells within specified cortical layers via anatomically oriented minicolumns. Results reveal target-specific, spatially tuned firing between inter-laminar (layer 2/3 and layer 5) pairs of neurons participating in the gating of information during the decision making phase of the task with differential correlations between activity in layer 2/3 and layer 5 in the integration of spatial vs. object-specific information for correct task performance. Such inter-laminar processing was exploited by the interfacing of an online model which delivered stimulation to layer 5 locations in a pattern associated with successful performance thereby closing the columnar loop externally in a manner that mimicked normal processing in the same task. These unique technologies demonstrate that PFC neurons encode and process information via minicolumns which provides a closed loop form of “executive function,” hence disruption of such inter-laminar processing could form the bases for cognitive dysfunction in primate brain.

## Introduction

The prefrontal cortex (PFC) with its privileged position at the top of sensory-motor processing hierarchy (Alexander et al., 1986; Fuster, 2001) has been traditionally viewed as the seat of higher cognitive functions such as working memory and executive control of behavior (Fuster and Alexander, 1971; Funahashi et al., 1989; Miller, 2000). According to many theories of cognition, cortical mechanisms of executive function coordinate and control “online” cognitive processes underlying memory storage, behavioral selection and motor planning (Posner and Snyder, 1975; Goldman-Rakic, 1996; Shallice and Burgess, 1996; Miyaki et al., 2000; Miller and Cohen, 2001; Baddeley, 2002; Graybiel, 2008). Prefrontal neural activity in the primate brain that emerges from cortical laminar minicolumns is hypothesized to play a critical role in cognitive processes dealing with working memory and executive control of behavior (Goldman-Rakic, 1996; Mountcastle, 1997; Rao et al., 1999; Miller and Cohen, 2001; Baddeley, 2002; Casanova et al., 2007, 2009).

Cortical minicolumns consist of vertically-oriented “aggregates” of cell bodies that represent the basic anatomic and physiologic microcircuitry of the cerebral cortex (Mountcastle, 2003) that consist of pyramidal cells and several types of GABAergic, inhibitory interneurons (i.e., double-bouquet, basket, and chandelier cells) (Casanova et al., 2002a,b, 2007; Sokhadze et al., 2012). Minicolumns in PFC are interconnected to each other through horizontal “long range” projections in layer 2/3 (Kritzer and Goldman-Rakic, 1995), inter-laminar mini-loops (Weiler et al., 2008; Takeuchi et al., 2011) and “reverberatory loops” through projections to the subcortical basal ganglia nuclei and thalamus (Alexander et al., 1986). Such “reverberatory loops” combine incoming signals from thalamus in layer 4 and inputs from cortical horizontal projections in layer 2/3, in order to compare inputs to a threshold criterion triggering an output response under specific conditions.

The ability to make behavioral selections in humans involves attention, target/goal choice, planning and monitoring of actions, and is regarded as a facet of decision making based on sensory evidence, expected costs, and benefits associated with the outcome (Opris and Bruce, 2005; Opris et al., 2005a,b; Heekeren et al., 2008; Pesaran et al., 2008; Resulaj et al., 2009). In order to make optimal selections or decisions, many areas in the primate brain with converging inputs to the supra-granular layers of the PFC are activated (Kritzer and Goldman-Rakic, 1995; Opris et al., 2011; Takeuchi et al., 2011), thus raising the question as to how the PFC processes information required for selection of a particular behavioral response necessary for achieving functional objective. It has been shown that neurons in PFC recorded from rhesus macaque nonhuman primates (NHPs) demonstrate functional interactions between inter-laminar “cell pairs” synaptically connected via cortical minicolumns (Kritzer and Goldman-Rakic, 1995; Mountcastle, 1997; Buffalo et al., 2011; Opris et al., 2011; Takeuchi et al., 2011) and that these cells coordinate activity required to encode spatial location and select the target location or target features.

In the studies presented here this presumed executive function of PFC minicolumns was examined via custom designed conformal multielectrode arrays (MEAs) implemented to record the firing of inter-laminar cell pairs oriented in cortical “microstrips” in NHPs (Opris et al., 2011). The recording pads on the MEAs matched the dimensions of two interconnected cell layers in PFC (layer 2/3 and layer 5) which allowed simultaneous monitoring of columnar oriented cells in each layer in order to characterize the control of arm movements in a cognitive task requiring working memory and image-based target selection (Deadwyler et al., 2007; Hampson et al., 2011). The results reveal target-specific, spatially tuned firing between columnar oriented pairs of inter-laminar PFC neurons, during the decision making and/or motor planning phase of the task (Hampson et al., 2011).

## Methods

All animal procedures were reviewed and approved by the Institutional Animal Care and Use Committee of Wake Forest University, in accordance with U.S. Department of Agriculture, International Association for the Assessment and Accreditation of Laboratory Animal Care, and National Institutes of Health guidelines.

### Visual delayed-match-to-sample (DMS) task

**Figure 1:**
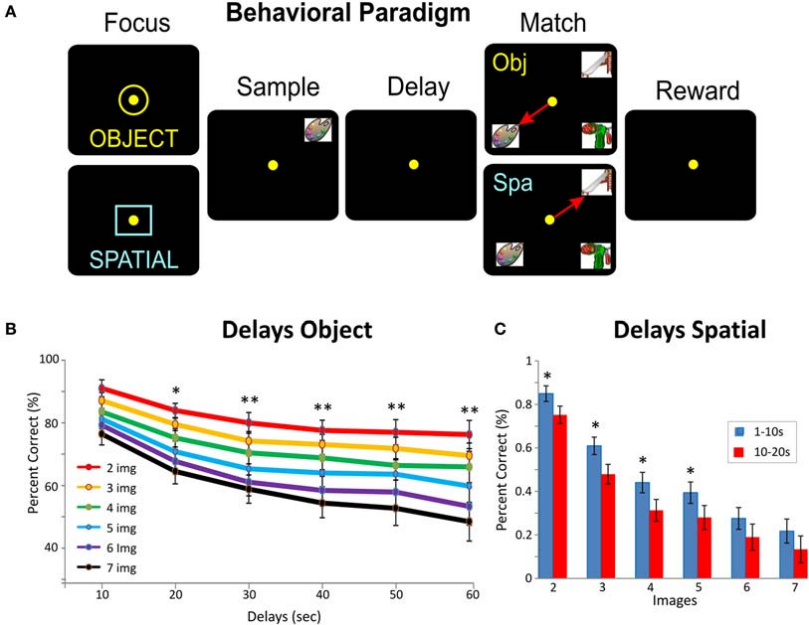
**Delayed match to sample task (DMS) in NHPs. (A)** Behavioral paradigm shows the DMS task in which two types of trials (*Object* of *Spatial*) were signaled by presentation of one of the two “Focus” signals into which the animal placed the cursor to start the trial. On *Object* trials (yellow ring) reward was delivered for selection of the same clip-art image to be presented in the Sample phase, when it appeared later in the Match phase of the trial, irrespective of position on the screen. On *Spatial* trials (blue square) reward was delivered in the Match phase for selection of the image in the “spatial location on the screen” in which the image was presented in the Sample phase, irrespective of the clip-art image occupying that position in the Match phase. The sequence of events on both types of trials: (1) presentation of “Focus signal” to initiate the trial with cursor placement into the signal, (2) presentation of the ‘Sample’ clip-art image requiring cursor movement into the image “Sample Response” (3) initiation of a variable “Delay” interval of 1–60 s with the screen blank, (4) upon timeout of the delay interval the Match phase is initiated in which the Sample image is presented on the screen at random locations accompanied by 1–6 other non-match (distracter) images. On *Object* trials placement of the cursor into the (Sample) image for = 0.5 s was the correct Match response (MR) for that trial type. On *Spatial* trials placement of the cursor into the same position in which the image appeared in the Sample phase of the trial was the correct MR. Both correct MRs produced a juice reward via a sipper tube mounted next to the animal's mouth. Placement of the cursor into an inappropriate image or location for = 0.5 s caused the trial to terminate and the screen to blank without reward delivery. The inter-trial interval (ITI) was 10.0 s, and Object and Spatial trials were randomly presented 0.6 and 0.4 percent of trials per session, respectively. **(B)** DMS performance averaged over all animals (mean % correct MRs) for *Object* trials as a function of number of Match phase distracter images (number of images 2–7) and length of delay interval (10–60 s) Asterisks: ^*^F_(1, 486)_ = 7.98, *p* < 0.01, ^**^F_(1, 486)_ = 12.24, *p* < 0.001, ANOVA. **(C)** Behavioral performance averaged over all animals (mean % correct MRs) for *Spatial* trials as a function of length of delay interval (1–20 s) and number of Match phase distracter images (number of images 2–7) Asterisks: ^*^F_(1, 486)_ = 7.98, *p* < 0.01, ANOVA.

The NHPs utilized as subjects in this study (*n* = 4) were trained for at least 2 years to perform a well characterized, custom-designed visual delayed-match-to-sample (DMS) task (Hampson et al., 2011; Opris et al., 2011) shown in Figure 1A. Animals were seated in a primate chair with a platform in front of a display screen in which position of the arm on the platform was tracked via a UV-fluorescent reflector affixed to the back of the wrist, illuminated via a 15 W UV lamp, and detected by an LCD camera positioned 30 cm above. Hand position and movement was digitized and displayed as a bright yellow cursor on the screen and horizontal positions of illuminated clip-art targets were computed from the video image using a Plexon Cineplex scanner. The DMS task paradigm is shown in Figure 1A. Trials were initiated by the animal placing the cursor inside a yellow 3” circle or square randomly illuminated in one of the nine spatial positions on the screen. The presence of either the circle or square constituted the “Start” signal for the trial and indicated “trial type” with respect to the Match reward contingency on the same trial (Figure 1A). Placement of the cursor into the Start signal image produced a trial unique clip-art image randomly displayed in one of eight peripheral screen positions on each trial for 2, 0 s, which characterized the “Sample Phase” of the task. Movement of the cursor into the Sample image (Sample Response) blanked the screen and initiated the Delay phase for 10–60 s, randomly selected on each trial. Timeout of the Delay interval initiated the onset of the Match phase of the task (Match phase “onset”) in which 2–7 trial unique clip-art images, including the Sample image, were presented on the screen with position selected randomly on each trial. Placing the cursor into either, (1) the Sample image (*Object* trial) or (2) the same location as the prior Sample Response (*Spatial* trial), during the Match phase constituted the correct “Match Response (MR)” which produced a drop of juice as the reward, delivered via a sipper tube located near the animal's mouth, and blanked the screen for 10 s until the next trial. Placement of the cursor into one of the non-match (distracter) images on an *Object* trial, or a different spatial location on the screen during a *Spatial* trial, constituted a MR error that blanked the screen without reward delivery and initiated the 10 s inter-trial interval (ITI). All clip-art images (sample and distracter) were unique for each trial in sessions of 100–150 trials and were chosen from a 10,000 image selection buffer which was updated to replace 20% of the images every month. The four NHPs were trained to overall performance levels of 70–75% correct with respect to the above described DMS task parameters.

### Surgery

**Figure 2:**
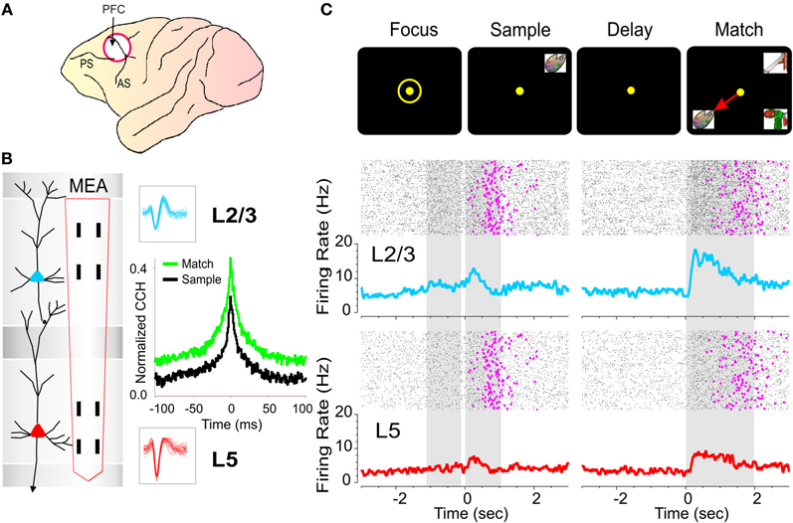
**Inter-laminar recording in primate prefrontal cortex during delayed-match-to-sample (DMS) task. (A)** Diagram of NHP brain showing PFC recording locations in cortical areas 46, 8, 6 (white circle). **(B)** Recording Array: Diagram of a cortical minicolumn consisting of a “columnar pair” of L2/3 (blue) and L5 (red) PFC cells. Diagram shows conformal multielectrode recording array (MEA) positioned for simultaneous inter-laminar columnar recording from a PFC minicolumn with corresponding L2/3 and L5 cell waveforms (blue and red) corresponding to the task specific neural firing shown in **(C)**. **(C)** Individual trial rasters and average perievent histograms (PEHs) over 50 trials obtained from the inter-laminar “cell pair” recorded simultaneously from L2/3 (blue) and L5 (red) in the minicolumn format shown in **(B)** over ±2.0 s relative to both the Sample and Match phase onset (0.0 s) in a single DMS session. The occurrence of behavioral responses (reaction time plus movement time) in the Match phase on each trial is indicated by pink dots in the rasters. **(B,C)**: Cross-correlation histograms (CCHs) of L2/3 and L5 cell pair activity in **(C)** from the same cell pair in **(B)** for the Sample and Match phases of the task. The larger “green” CCH peak shows increased inter-laminar synchronization during target selection in the Match phase relative to the CCH constructed from the same cell pair during the Sample phase (black) of the task shown in **(C)**.

Animals were surgically prepared with cylinders for attachment of a microelectrode manipulator over the specified brain regions of interest. During surgery animals were anesthetized with ketamine (10 mg/kg), then intubated and maintained with isoflurane (1–2% in oxygen 6 l/min). Recording cylinders (Crist Instruments, Hagerstown, MD) were placed over 20 mm diameter craniotomies for electrode access to stereotaxic coordinates of the Frontal Cortex (25 mm anterior relative to interaural line and 12 mm lateral to midline/vertex) in the caudal region of the Principal Sulcus (Figure 2A), the dorsal limb of Arcuate Sulcus in area 8 and the dorsal part of premotor area 6 (Hampson et al., 2011), areas previously shown by PET imaging to become activated during task performance (Hampson et al., 2009). Two titanium posts were secured to the skull for head restraint with titanium steel screws embedded in bone cement. Following surgery, animals were given 0.025 mg/kg buprenorphine for analgesia and penicillin to prevent infection. Recording cylinders were disinfected thrice weekly with Betadine during recovery and daily during recording. Vascular access ports (Norfolk Medical Products, Skokie, IL) for drug infusions were implanted subcutaneously in the mid-scapular region, the end of the catheter threaded subcutaneously, to a femoral incision, inserted into the femoral vein, and threaded for a distance calculated to terminate in the vena cava and flushed daily with 5 ml heparinized saline needed for IV drug administration.

### Electrophysiology: recording and stimulation

Electrophysiological procedures and analysis utilized the MAP Spike Sorter by Plexon, Inc. (Dallas, TX) for 64 channels simultaneous recordings. Customized conformal designed ceramic MEAs were constructed at the University of Kentucky, Center for Microelectrode Technology—CenMet, Lexington, KY, and consisted of etched platinum pads (Figure 2B) for recording multiple single neuron activity (Hampson et al., 2004, 2011). Single extracellular action potentials (Figure 2B) were isolated and analyzed with respect to activity on specific recording pads (mpedance range 0.5–3.0 MOhms) during different events within DMS trials (Figure 2C). The configuration of the MEA (Figure 2B) was specially designed to conform to the columnar anatomy of the PFC such that the top four recording pads recorded activity from neurons in the supra-granular layer 2/3 (L2/3) while the lower set of four pads, separated vertically by 1350 μm, simultaneously recorded neuron activity in the infra-granular layer 5 (L5) of the PFC (Figures 2B and C). Recordings from multiple pads in designated locations on the MEAs were analyzed by a nonlinear model previously perfected for assessing and extracting spatiotemporal multineuron firing patterns in PFC using the same MEAs and to deliver task-contingent electrical stimulation to L5 in the same pattern as recorded during correct trial performance (Hampson et al., 2012). Stimulation consisted of 1.0 ms bipolar pulses (50–70 uA) delivered to L5 recording locations following presentation of the Match phase screen and prior to the completion of the MR (Figure 7).

### Electrochemical recording

Ceramic MEAs similar to those utilized above for electrophysiological recording were also prepared for electrochemical recording (Burmeister et al., 2004, 2008; Quintero et al., 2007, 2011; Hascup et al., 2008, 2011; Fuqua et al., 2010). The electrochemistry arrays consisted of four recording sites (15 × 333 μM) in two rows, separated by 500 μm, with a 7 cm polyimide shaft for depth positioning. The electrodes were configured to record from Layer 2/3 with the reference in Layer 1. MEAs were dip coated with Nafion^®^, a fluoropolymer which excludes the passage of anions, thus ensuring that only cations would reach the platinum recording surface. The dorsal (“sentinel” or reference) recording sites were coated with bovine serum albumin (BSA) plus glutaraldehyde; ventral recording sites were coated with Glutamate oxidase and BSA + glutaraldehyde. The GluOx coating allowed the ventral pads to be sensitive to glutamate release through the enzymatic production of H_2_O_2_. A +0.7V charging potential was applied to the MEA once per second (using an Ag/AgCl reference electrode) to oxidize the H_2_O_2_ resulting from detection of glutamate at the electrode. The “relaxation” current from H_2_O_2_ oxidation was proportional to second-by-second changes in glutamate concentration at the electrode (Quintero et al., 2011).

### Data analysis

**Figure 3:**
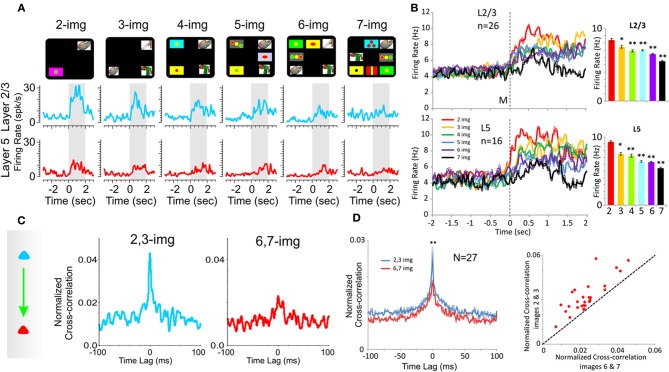
**Effect of number of images on PFC columnar firing. (A)** Example peri-event histograms comparing neuron firing in PFC layers L2/3 (blue) and L5 (red) as a function of the number of images presented (upper: display screens) in the Match phase on *Object* type trials in the DMS task. **(B)** Population peri-event histograms depicting the activity of prefrontal cells from layers L2/3 (*n* = 16) and L5 (*n* = 26) on all types of trials with different numbers of images (2, 3, 4, 5, 6, and 7) presented during match phase in the DMS task [L2/3: *F*_(6, 1039)_ = 8.29, *p* < 0.001; L5: *F*_(6, 639)_ = 8.64; *p* < 0.001, ANOVA]. **(C)** Example inter-laminar CCHs for trials with a few (2 and 3 images) vs. many (6 and 7 images) distracter images constructed from the same interlaminar L2/3-L5 cell pair shown in **(A)**. **(D)** Normalized population CCHs for trials with low (2, 3 red) vs. high (6, 7 blue) numbers of images in the Match phase consisting of the average correlation coefficients across individual CCHs from 27 different inter-laminar cell pairs. Scatter plot showing differential distributions of individual CCH peak correlation coefficients on trials with low vs. high numbers of images for the same cell pairs (*n* = 27) comprising the population CCH. ^**^p < 0.001, ^*^p < 0.01, ANOVA.

**Figure 4:**
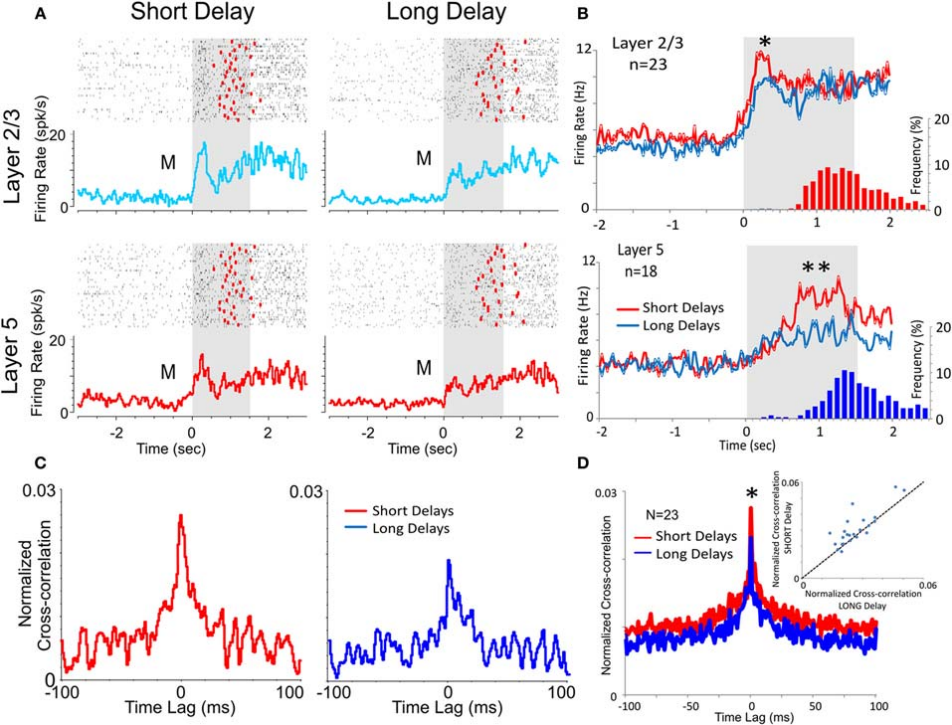
**Effect of DMS delay duration on PFC neuronal activity and columnar firing. (A)** Raster and peri-event histograms comparing firing of PFC L2/3 and L5 cells in the Match phase as a function of short (≤20 s) vs. long (>40 s) delays in the DMS task. **(B)** Population peri-event histograms depicting the activity of L2/3 and L5 cells during short (≤20 s) vs. long (>40 s) delays (*n* = 23 cells in L2/3, *p* < 0.01 ANOVA and *n* = 18 cells in L5; *p* < 0.001 ANOVA). Histograms show distribution of Match response latencies for short (red) vs. long (blue) delays. **(C)** Example inter-laminar CCHs for L2/3 and L5 cell pairs shown in **(B)** on trials with short vs. long delays. **(D)** Population of inter-laminar CCHs and scatter plot for short vs. long delay trials (see Figure 3D). ^**^p < 0.001, ^*^p < 0.01, ANOVA.

**Figure 5:**
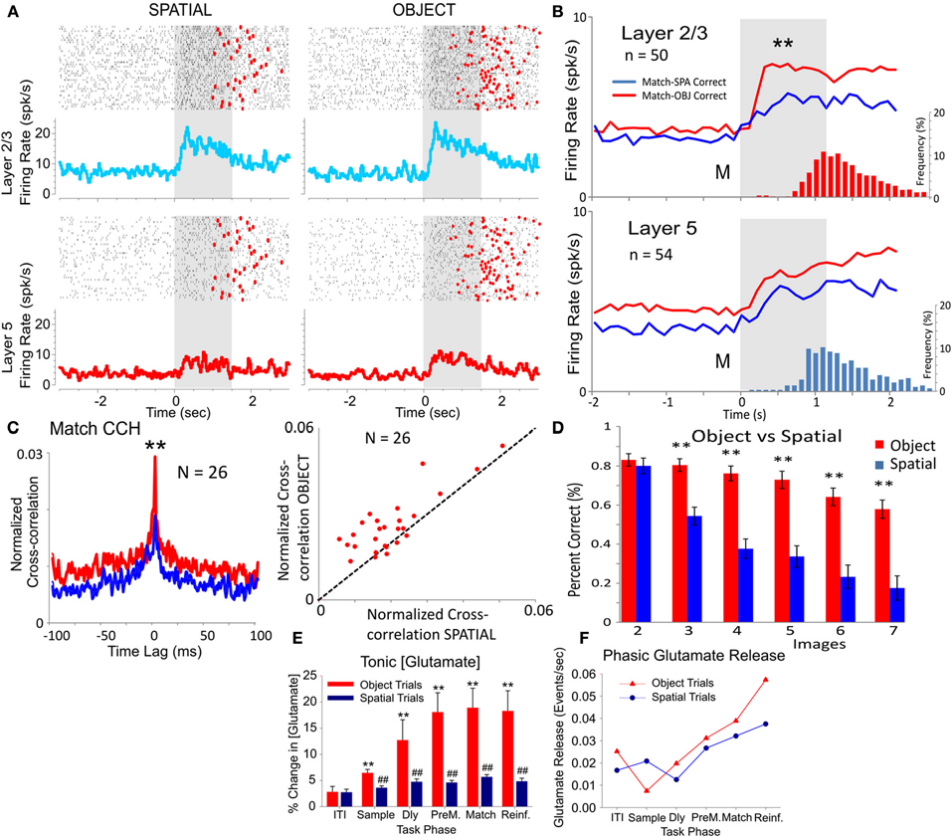
**Comparison of PFC inter-laminar firing on *Object* vs. *Spatial* trials. (A)** Rasters and Peri-event histograms showing firing in the Match phase of a PFC L2/3-L5 cell pair recorded during *Spatial* vs. *Object* type trials. **(B)** Population peri-event histograms depicting the activity of PFC L2/3 (*n* = 50) and L5 (*n* = 54) cells on *Spatial* (blue) vs. *Object* (red) trials presented during match phase in the DMS task [*F*_(1, 1039)_ = 12.89, *p* < 0.001, ANOVA]. Histograms show distribution of match response latencies for *Spatial* (blue) vs. *Object* (red) trials. **(C).** Average inter-laminar cross-correlation for the same cell pairs (*n* = 26) recorded on *Object* vs. *Spatial* trials. Scatter plot of shows differential distribution of peak CCH values for *Object* vs. *Spatial* trials for the same cell pairs. **(D)** Behavioral performance as a function of the number of images (2–7) on *Object* vs. *Spatial* trials. **(E)** Electrochemical recording of tonic glutamate neurotransmitter concentrations in PFC Layer 2/3. Mean (±S.E.M.) glutamate concentration ([Glutamate]) measured as a percentage increase over baseline (average 8.69 ± 0.77 μM) glutamate concentration. Horizontal axis indicates phase of DMS task: intertrial interval (ITI), Sample phase, Delay phase (Dly), end of delay phase 5 s prior to Match (PreM), Match phase and reinforcement (Reinf.). Asterisks: ^*^p < 0.01, ^**^p < 0.001, *Object* vs. *Spatial* trials; ^#^*p* < 0.01, ^##^*p* < 0.001 DMS task phases vs. ITI. **(F)** Frequency of phasic glutamate release events measured as transient increase (<2.0 s duration) of at least 5% in [Glutamate] for the same trials shown **(E)**. Frequency normalized to number of events per second per DMS trial (Fuqua et al., 2010).

Task performance was determined for each animal (*n* = 4) as percent correct trials within and across sessions and related to simultaneous MEA recordings on individual trials during Match phase image selection MR in the task (Hampson et al., 2011). Cell types were identified as regular firing pyramidal cells in terms of baseline (nonevent) firing rate (Opris et al., 2009) and significant changes (*z* > 3.09, *p* < 0.001) in firing (see below) on single trials in perievent histograms (PEHs) derived for intervals of ±2.0 s relative to the time of Match screen presentation that signaled onset of the Match phase of the task (Figure 2C). Task-related neural activity was classified according to locations on the conformal MEA which were positioned specifically in L2/3 and L5 (Figure 2B) upon insertion in PFC prior to the start of the DMS session. To account for neuronal responses in terms of columnar microcircuit organization neurons recorded on the MEAs were characterized by (1) simultaneous cell activity on both sets of vertical separated (1350 μm) pads (L2/3 cell upper and L5 cell lower), during electrode positioning (Figure 2B), and (2) whether the same cell pair firing was modulated similarly during the Match phase of the DMS task (Hampson et al., 2012). Standard (Z) scores of increased firing rates relative to nonevent baseline values were calculated for individual cells for each DMS task event. Firing rate was analyzed in 250 ms bins for ±2.0 s relative to time of initiation (0.0 s) task events. Only neurons with firing rates significantly elevated from that in pre-event phases (−2.0 to 0.0 s) baseline period were included for analysis. Differences in cross-correlation between neuron spikes of L2/3 and L5 cell pairs on the same vertical sets of MEA pads (Figure 2B) were assessed for the same temporal intervals using standardized distributions of correlation coefficients assessed under different conditions related to performance in the Match Phase (Figures 2–5). Mean cross-correlation histograms (CCHs) were calculated and compared relative to mean coefficients normalized relative to probability of firing for the same populations of cell pairs under different experimental conditions (Figures 2B, 3C, 4C, and 5C), all of which satisfied the 99% confidence requirement (Opris et al., 2011). CCHs were generated using a shift predictor algorithm built into NeuroExplorer version 4 (http://www.neuroexplorer.com/), which computed chance cross-correlation levels by randomizing the actual spike sequence and calculating cross-correlations five different times for a given pair of neurons, which was then subtracted from the true coefficients for CCHs to adjust for correlated firing due to differences in cell firing rates and frequency of bursting (Opris et al., 2011; Takeuchi et al., 2011). Population (mean) CCHs, normalized as a function of probability, were computed by averaging coefficients across multiple cell pairs and plotting the mean values (±SEM) in 1.0 ms bins (Figures 2B, 3C, 4C, and 5C).

### Identification of cortical layers and minicolumns

The conformal MEA (model W3) probe (Figure 2B) was designed so that the two sets of recording pads could only record simultaneous activity from neurons separated by ~1350 μm, which given the orientation of insertion into PFC (Figure 1B) could only consist of infra-granular layer 5 and supra-granular layer 2/3 cell activity (Hansen and Dragoi, 2011; Opris et al., 2011; Takeuchi et al., 2011). Misplacement of the probe due to a different angular penetration relative to columnar orientation in PFC was detectable by the absence of simultaneous cell recordings on the sets of vertically separated (1350 μm) pads. In addition, the MEA (Hampson et al., 2004; Opris et al., 2011) employed here allowed simultaneous recording of two PFC minicolumns (Figure 2B) since, with proper vertical alignment (<5.0°), activity from adjacent minicolumns could be detected, since MEA pads were separated laterally by 40 μm which exceeds the distances reported (28 μm) from anatomic assessments (Casanova et al., 2009; Hansen and Dragoi, 2011; Mo et al., 2011; Takeuchi et al., 2011).

### Tuning plots

**Figure 6:**
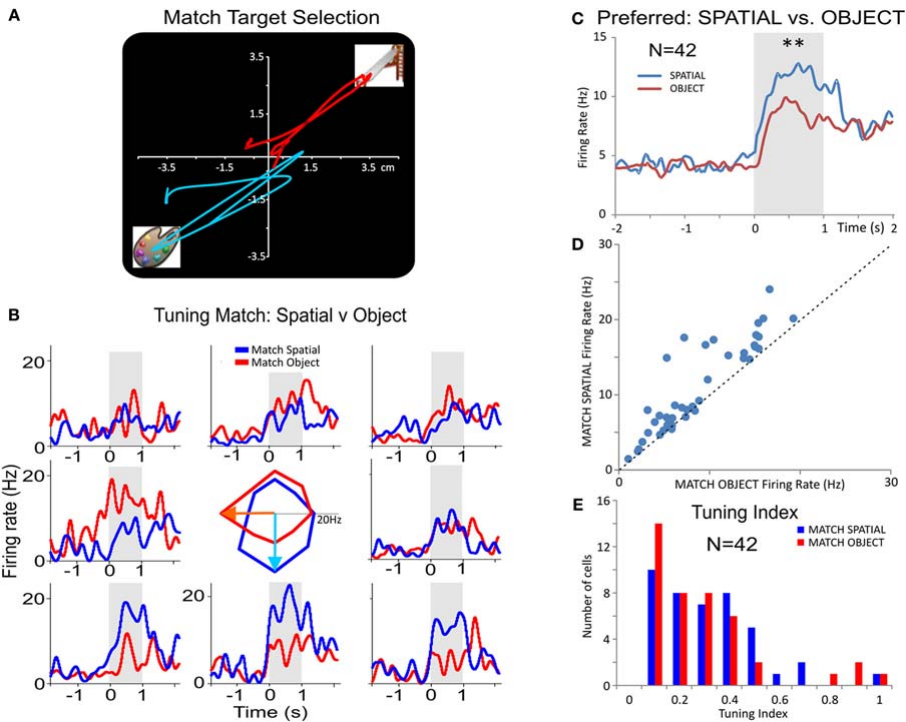
**Inter-laminar PFC spatial tuning during DMS task performance. (A)** Illustration of two arm movement tracks from a single animal for two opposite target locations on the screen during the Match phase of the DMS task. **(B)** Tuning Plot Multigram PEHs (multigram) and spatial tuning plot (diagram in center) for a PFC L2/3 cell on *Spatial* (blue) and *Object* (red) trials. The tuning plot in the middle displays Match phase mean firing rates (shaded areas in PEHs) along radial axes corresponding to movement of the cursor into each of the eight screen image positions from the screen center summed over all trials in a single session. The spatial (i.e. screen position) “bias” indicated by the highest firing rate for target selection, for the *Object* trial tuning vectors was in the “medial left” position (i.e.,180°), while the bias for *Spatial* trial tuning vectors was in the “down” (i.e., 270°) position. **(C)** Average firing rate for *Spatial* biases (preferred target locations) and Object biases summed across different (*n* = 42) inter-laminar (L2/3 and L5) cells. **(D)** Scatter plot comparing preferred (i.e., highest) firing rate directions for the same cells in **(C)** on *Spatial* vs. *Object* trials, indicating a more biased directional firing on *Spatial* trials. **(E)** Histogram comparing the distribution of preferred firing for the same cells as a function of a tuning index (TI) derived as TI = (PF−NF)/(PF+NF), on *Spatial* (blue) and *Object* (red) trials, where PF represents preferred location/direction firing rate and NF stands for non-preferred direction firing rate. The plot in **(E)** shows that there was a trend for lower TIs, less bias for one position, on *Spatial* vs. *Object* trials by showing more cells with lower TI values. ^**^p < 0.001, ANOVA.

For each inter-laminar cell pair (L2/3 and L5), firing on the same trials was plotted with respect to the position of the target selected in the Match phase (Figure 6B). Directionality was assigned according to the eight positions on the screen with reference to placement of the cursor in the center providing angles corresponding to the location of the match image around the periphery of the screen, yielding 0° (directly lateral), 45, 90, 135, 180, 225, 270, 315, and 360° movement directions from center of screen (Rao et al., 1999; Felsen et al., 2002). Mean firing rate commencing at Match phase onset until time of occurrence of the MR (i.e., typically 0.5–1.0 s, Figures 4D and 5D) was calculated and represented for each inter-laminar cell pair in polar coordinates as tuning plots of the average firing rate, over all trials in a single session. Directional biases, or “preferences”, for cell pairs were defined as response locations with the highest mean firing rates relative to all the other positions responded to during the session (Figure 6B). A *tuning index* plot (Meyer et al., 2011) was employed for comparing the distribution of biases for the same cells on *Object* vs. *Spatial* trials (Figure 6E).

## Results

The four subjects NHPs trained to perform the DMS task (Hampson et al., 2011) were required to select the same video image presented on-screen in the prior Sample phase from a set of 2–7 images in the subsequent Match Phase after an intervening Delay of 10–60 s (Figure 1A). The NHPs made hand tracking movements of a cursor on the screen in the Match phase to obtain a juice reward for selection of the correct (Sample) image in different positions which varied on each trial with respect to image-type and screen position. The key variables in the task therefore were: (1) number of distracter images (2–7) presented randomly in different screen positions in the Match phase on each trial, (2) the duration of the intervenning delay interval (1.0–60.0 s) and (3) the random placement of the Sample (target) image in 1 of 7 spatial positions on the screen in the Match phase (after the delay interval). Previous research with the same DMS task has indicated the necessity of attention, short-term memory and response latency, together with influence of type of choice, as factors that affect cognitive workload in the same task (Porrino et al., 2005; Deadwyler et al., 2007). Recent analyses of PFC activity showed that animals execute a “decision process” in the Match phase of the task (Figure 1A) involving target selection (Hampson et al., 2011) and that this involved inter-laminar synchrony in cell activity (Hampson et al., 2012). In the study presented here PFC columnar inter-laminar pair-wise cell firing from four NHPs (60 cell pairs: 21 in animal K, 16 in B, 12 in E and 11 in G) was characterized for all of the above mentioned task-related parameters shown previously (Porrino et al., 2005; Deadwyler et al., 2007) to control cognitive processing in this DMS task.

### Multielectrode array recordings from cortical layers and minicolumns

Prior reports of neural relationships to executive function and decision making in a sensorimotor hierarchy (Miller and Cohen, 2001; Opris and Bruce, 2005; Heekeren et al., 2008; Pesaran et al., 2008; Opris et al., 2012) referred to recordings made in dorsolateral PFC as shown in Figures 2A and B, which were also reported to depend on the interaction between neurons in different layers in the same area (Goldman-Rakic, 1996; Opris et al., 2011; Takeuchi et al., 2011). In this study, inter-laminar connectivity was sensed by previously described conformal-designed MEAs (Hampson et al., 2012) positioned to simultaneously record neurons located in PFC layer 2/3 and layer 5 in adjacent “minicolumns” during performance of the DMS task (Figures 2B and C). The MEA contained two linear sets of four recording pads separated vertically by 1350 μm to conform to the distance between PFC cortical cell layer 2/3 (L2/3) and layer 5 (L5) when inserted perpendicular to the parallel lamellae (see “Methods”). The two sets of dual vertical pads in each upper and lower position on the MEA were separated horizontally by 40 μm in order to exceed the reported 28 μm width of single cortical minicolumns (Casanova et al., 2007; Opris et al., 2011). This allowed simultaneous recording from two adjacent L2/3 and L5 columnar “cell pairs” constituting neural activity from two separate minicolumns on a single MEA probe. This pad configuration insured that only cells in L2/3 and L5 were recorded, since the appearance of cells simultaneously on both sets of vertical pads required 0° angular placement relative to both cell layers (Takeuchi et al., 2011) as shown in Figure 2B. In this study spatiotemporal analyses of 180 prefrontal cortical (PFC) pyramidal cells recorded in four NHPs revealed a large number (*n* = 60) of confirmed L2/3 and L5 cell pairs in this region of PFC (Figure 2A) that displayed inter-laminar interactions during the Match phase of the DMS task.

### Inter-laminar processing in PFC during DMS task

The relevance of minicolumnar activity to decision making has been investigated in several types of cognitive processing tasks (Goldman-Rakic, 1996; Opris and Bruce, 2005; Heekeren et al., 2008; Pesaran et al., 2008; Resulaj et al., 2009; Opris et al., 2011, 2012). An example of this inter-laminar interaction during the target-selection in the Match phase of the DMS task (Figure 1A) is shown in Figure 2C in raster/PEHs constructed over ±2.0 s for the Sample and Match phases of the trial for a cell pair recorded in the PFC with the MEA (Figure 2B). The cell pair was recorded on appropriate sets of pads as shown in the illustration of the two cells in L2/3 and L5 next to the MEA (Figure 2B). Neurons in both layers showed significant increases in mean firing during Sample (L2/3: *Z* = 7.30, *p* < 0.001; L5: *Z* = 4.16, *p* < 0.001) and Match (L2/3: *Z* = 12.86, *p* < 0.001; L5: *Z* = 6.20, *p* < 0.001) screen presentations (post events: 0.0–2.0 s) and during subsequent movements associated with target selection in this task (Hampson et al., 2011). A consistent finding employing this recording configuration was that within neuron pairs significantly higher mean firing rate in the 0.0 + 2.0 s interval were observed for L2/3 cells after Match phase onset [*F*_(1, 153)_ = 20.93, *p* < 0.001] as demonstrated in the upper and lower raster/PEHs in Figure 2C. More precise functional connections between individual cells within each minicolumn was determined by cross (CCHs; Opris et al., 2011; Takeuchi et al., 2011; Hong et al., 2012) constructed for the same minicolumn cell pairs. This is shown for the firing displayed in the PEHs in Figure 2C and although there was significantly correlated firing (Match: *Z* = 12.23, *p* < 0.001; Sample: *Z* = 10.12, *p* < 0.001) the differences in peak correlation shown in the CCHs [*F*_(1, 401)_ = 9.41, *p* < 0.001] indicate that the cell pair firing was more synchronized in the Match than in the Sample phase of the task.

### Effects of task difficulty on inter-laminar processing

#### Number of match phase images

As shown in prior reports (Porrino et al., 2005; Deadwyler et al., 2007; Hampson et al., 2011) a major cognitive factor influencing target selection in the Match phase of this task was the number of distracter images (number of images) presented with the Sample image on a given trial (Figure 1A). Figure 3A shows an example of a graded decrease in cell pair firing in both L2/3 and L5 as a function of the number of images presented in the Match phase. In agreement with prior results (Hampson et al., 2011), overall mean firing rates of L2/3 (*n* = 26) and L5 (*n* = 16) neurons (Figure 3B) were systematically decreased as a function of the number of images in the Match phase (L2/3: *F*_(6, 1039)_ = 8.29, *p* < 0.001; L5: *F*_(6, 639)_ = 8.64; *p* < 0.001, ANOVA). However, more importantly this decrease was also expressed in terms of correlated firing between L2/3-L5 cell pairs as shown in Figures 3C and D (*n* = 27) in which Match phase CCHs on trials with few (2 and 3) images showed significantly higher correlations than on trials with more (6 and 7) images [*F*_(1, 53)_ = 7.21; *p* < 0.01, ANOVA]. This finding of decreased inter-laminar correlated firing is consistent with the fact that increasing the number of distracter images decreases task performance (Figures 1B and C) due to an increase the in cognitive workload of the task (Hampson et al., 2011; Kelley and Lavie, 2011).

#### Duration of delay

Another factor increasing cognitive workload in the DMS task is memory of the Sample target image across the delay interval (Figure 1A) and has been shown to be a factor influencing Match target selection (Deadwyler et al., 2007). Consistent with this relationship as shown in Figure 4B was the fact that average firing rates for L2/3 and L5 cell pairs was significantly lower on “long” (>40 s) vs. “short” (=20 s) delay trials [L2/3: *F*_(1, 919) = 6.67,_ *p* < 0.01, *n* = 23; L5: *F*_(1, 719)_ = 10.92; *p* < 0.001, *n* = 18, ANOVA]. Figure 4C shows that Match phase (0.0–2.0 s) CCHs for both L2/3 and L5 cells were significantly lower on “short” vs. “long” delay trials [short delay: *F*_(1, 1639)_ = 10.87, *p* < 0.001; long delay: *F*_(1, 1639)_ = 6.71, *p* < 0.01] as were the average CCHs for all L2/3 vs. L5 cell pairs (Figure 4B) under both conditions [*F*_(1, 45)_ = 7.27; *p* < 0.01, ANOVA]. The decrease in interlaminar correlation as a function of short vs. long delays is shown more explicitly in the scatterplot in Figure 4D where short delay trials produced higher correlation coefficients than long delay trials for the same cell pairs.

### Effect of ‘trial type’ (*object* vs. *spatial*) on inter-laminar processing

PFC minicolumns are a functional neuronal “module” (Buxhoeveden and Casanova, 2002; Casanova et al., 2003) with basic associative abilities to integrate horizontal and vertical anatomic “components” of the cortex (Mountcastle, 1997; Lund et al., 2003; Tanaka, 2003; Opris et al., 2011). The visual signals carrying *Spatial* information ascend from visual cortex on the dorsal stream to be integrated in PFC minicolumns with signals from the ventral stream that label the clip art image visual features such as color, shape, brightness used on *Object* trials. To compare firing in PFC layers L2/3 and L5 on *Spatial* vs. *Object* trials we examined image selection ability of cortical minicolumns during the Match phase of DMS task in the same cells during both types of trial in the same session. Figures 5A and B show differences in L2/3 and L5 cells with respect to mean (±SEM) firing rate changes during the Match phase interval of *Spatial* and *Object* trials trials within the same DMS sessions. Mean firing rates during the Match phase (0.0–2.0 s) were significantly higher for L2/3 vs. L5 cells for both types of trials [*F*_(1, 1039)_ = 12.89, *p* < 0.001, ANOVA], however, Figure 5B shows that rates were significantly lower on *Spatial* vs. *Object* trials for L2/3 cells [*F*_(1, 499)_ = 10.96, *p* < 0.001, *n* = 50], but not for L5 [*F*_(1, 539)_ = 1.12, ns, *n* = 54; ANOVA]. Figure 5C shows that these differences in firing rates were also associated with significant decreases in mean CCHs for the same L2/3-L5 cell pairs on *Object* vs. *Spatial* trials [*F*_(1, 51)_ = 12.20, *p* < 0.001] which as indicated in the “Methods” section, were not due to alterations in firing rate *per se* (Hong et al., 2012). These results are consistent with the differences in degree of difficulty between *Object* vs. *Spatial* trials with respect to task performance, as shown in Figure 5D.

The contribution of different cellular networks for differential columnar processing on *Object* vs. *Spatial* trials was examined by employing electrochemical recording of glutamate levels in PFC Layer 2/3. Glutamate neurotransmission have been implicated in learning and memory (Dudkin et al., 2003; Riedel et al., 2003), therefore we hypothesized that changes in levels of released glutamate would correlate with differential cognitive processing (Stephens et al., 2010) of DMS trials. Glutamate-sensitive electrochemical recording MEAs were tested in three sessions for each of the four NHPs. The average basal glutamate concentration across animals and sessions was 8.69 ± 0.77 μM. Figure 5E shows the percent change in *tonic* glutamate concentration (Glutamate) from baseline sorted by individual phases (events) in the DMS task averaged separately across animals for *Object* vs. *Spatial* trials. While both *Object* and *Spatial* trials exhibited significantly increased glutamate concentrations [*F*_(5, 789)_ = 11.42, *p* < 0.001] in the Delay and Match phases of the task compared to baseline and ITI levels (Fuqua et al., 2010), glutamate levels were significantly elevated on *Object* relative to *Spatial* trials [*F*_(2, 789)_ = 32.17, *p* < 0.001]. Figure 5F depicts the frequency of *phasic* (i.e., transient) glutamate increases putatively related to neurotransmitter release events (Stephens et al., 2010). Although the frequency of glutamate release detected in the vicinity of the electrode was similar, it was still greater for *Object* vs. *Spatial* trials (Figure 5E) suggesting that the difference in overall tonic concentration represented activity of a network of glutamate synapses throughout PFC.

#### Spatial tuning

Another comparison of *Object* vs. *Spatial* trial processing was provided by examining “tuning plots” (Rao et al., 1999; Felsen et al., 2002) of PFC L2/3 and L5 cell pairs constructed for each target location on the screen during Match target selection (Figure 6A). Figure 6B shows an example of L2/3 cell firing on both *Spatial* (blue) and *Object* (red) trials. This type of comparison clearly dissociates the L2/3 cell biases/preferences on *Spatial*; tuning vector points to lower target location, 270°) vs. *Object* trials (Figure 6A; tuning vector points to left target location; 180°). Figure 6C shows average PEHs of preferred firing rates on *Spatial* (blue) vs. *Object* (red) trials for 42 neurons which showed significant increases [*F*_(1, 1679)_ = 19.63; *p* < 0.001, ANOVA] on *Spatial* vs. *Object* trials. Finally, a scatter plot of mean firing rates (Figure 6D) at biased target locations of the same cells (*n* = 42) as in Figure 6C shows a significant difference in preferred firing on *Spatial* vs. *Object* trials (*p* < 0.001; paired *T*-test). A “tuning index” defined as: TI =(PF−NF)/(PF+NF), where PF represents mean firing rate in the preferred/biased location and NF the non-preferred (lowest) firing location, was calculated to compare firing in the Match phase on *Object* vs. *Spatial* trials. Figure 6E shows the comparison of tuning index for Match target selection on *Spatial* vs. *Object* trials, that have comparable magnitudes in selection abilities on different, prior trial-specific instructions via the focus signal (Figure 1A), which is consistent with the multifunctional roles of these same cells in executive control. The results shown in Figures 6D and E indicate dominance of preferred location firing on *Spatial* vs. *Object* trials which was likely the result of the influence of the prior trial type instruction in the Focus phase of the task.

### Closing the loop with interlaminar regulated stimulation

**Figure 7:**
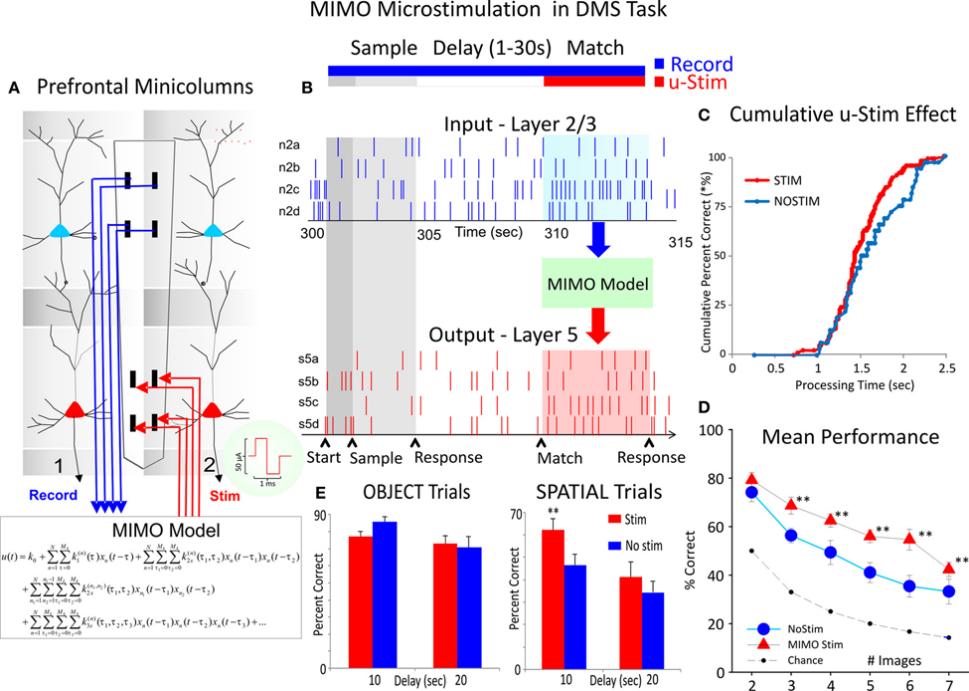
**Closing interlaminar loops in PFC with MIMO model generated stimulation. (A)** Diagram of the interfacing of MIMO model with conformal MEAs shown in Figure 2 between L2/3 and L5 during task performance. Electrical stimulation delivered to MEA pads in L5 via patterns of pulses (biphasic) recorded and derived from the same L5 locations on successful trials by the MIMO model. **(B)** Firing of L2/3 and L5 located columnar neurons as shown in Figure 2 recorded on line and fed to MIMO model shown in **(A)**. Shaded areas indicate time of Match Response execution during DMS trial, and the illustrated firing in L5 which is the same pattern as the delivered stimulation on trials with inappropriate L2/3 firing. **(C)** Changes in cummulative response latencies (processing time) from Match phase onset (“0”) during trials with stimulation delivered in the manner shown in (**A**) and (**B**), (**D**). Increase in performance across trials with increasing difficulty as a function of the number of Match phase distracter images on trials that received MIMO stimulation in the manner shown in **(A,E)**. Differential effects of MIMO stimulation on *Spatial* vs. *Object* trials showing more enhancement on *Spatial* trials ranging in delays of 1–20 s. ^**^p < 0.001, ANOVA.

The unique properties of conformal MEAs also provide the basis for applying a system specific model to control firing of cells via application of electrical stimulation to the same loci in which columnar firing has been detected and analyzed with respect to DMS task performance (Hampson et al., 2012). This same model was implemented here to test whether it could facilitate performance on trials that show a distinctive difference in performance as a function of the prior instructions as to type of response to make in the Match phase (i.e., *Object* vs. *Spatial* trials). Figure 7A shows the integration of a multi-input multi-output (MIMO) nonlinear math model to assess the patterns of firing in L2/3 and L5 cells recorded in the columnar manner with the MEA shown with adjacent vertical pads (Hampson et al., 2012; Opris et al., 2012). Figure 7B reflects the type of input and output firing patterns recorded and analyzed by the MIMO model and also illustrates how the output pattern of L5 cell firing is duplicated via a multichannel stimulator that is capable of delivering predetermined patterns of pulses to the same L5 pads to mimic firing on correct trials. The advantage of the MIMO model is that the online recording provides the means to detect when the inappropriate L2/3 firing pattern occurs which triggers the delivery of the appropriate L5 stimulation pattern providing the means to override errors and enhance performance (Hampson et al., 2012). The results of stimulation delivery are shown in Figures 7C and D, in which the effects on performance are compared to trials in which stimulation was not delivered, irrespective of trial type. Figure 7C shows the change in latency to respond on stimulation trials with respect to the time of onset of the Match phase, while Figure 7D shows the increase in correct performance on trials as a function of the number of distracter images in the Match phase. Finally in agreement with all prior demonstrations and correlations of columnar specificity with respect to the influence of trial type on DMS performance, Figure 7E shows that *Spatial* trials that received MIMO stimulation showed improved performance relative to *Object* trials (with the same number of distracter images and delays 1–20 s). These results indicate that MIMO derived stimulation facilitated cognitive processing required to retrieve the “rule” for successful Match phase selection of the appropriate Sample item as shown in Figure 6.

## Discussion

### Inter-lamnar processing in prefrontal cortex vs. closing the loop

The findings reported here (Figures 2, 3, and 4) are consistent with the idea that neurons in the supra- and infra-granular layers form efficient mini-columnar circuits during Match phase target selection required for effective performance of this DMS task (Swadlow et al., 2002; Pesaran et al., 2008; Resulaj et al., 2009; Buffalo et al., 2011; Opris et al., 2011; Takeuchi et al., 2011). The implementation of the unique MEA (Figure 2B) provided the basis for the detailed assessment of inter-laminar correlated firing (Opris et al., 2011) that was validated in multiple recordings of L2/3 and L5 cell pairs that yielded similar relations following differential changes in performance-dependent task parameters across animals and sessions (Figures 3D, 4D, and 5D). The increase in L2/3 and L5 correlations specific to the decision for target selection in the Match phase of the task (Figures 2, 3, and 4) suggests that a key variable in controlling task performance was activation of L5 neurons via specific minicolumnar input from paired neurons in layers 2 and 3 which have been shown to participate in the integration of “long-range” sensory inputs from the parietal dorsal visual stream (Opris and Bruce, 2005; Heekeren et al., 2008; Pesaran et al., 2008; Resulaj et al., 2009). Such integration was definitely reduced by trial difficulty as indicated by the reduction in firing synchrony between L2/3 and L5 cell pairs relative to trials with less cognitive demand (Figures 1B, 3C,D, and 4C,D). Prior investigations have shown that the firing of adjacent minicolumns is not correlated with respect to L2/3 and L5 activation during the Match phase of the task (Hampson et al., 2012; Opris et al., 2012). This again supports the notion that specific columnar processing was the basis for effective task performance and that such processing with respect to correlated firing between columns was independent, potentially reflecting processing of different forms of task specific information (Miyaki et al., 2000; Miller and Cohen, 2001; Opris et al., 2011).

Another feature demonstrating the columnar nature of this type of multineuron processing was the fact that classified L2/3–L5 cell pairs also showed the same Match phase spatial tuning biases (Felsen et al., 2002) during the session (Figure 6), which indicates the possible presence of previously identified PFC minicolumnar selection biases (Rao et al., 1999; Resulaj et al., 2009; Opris et al., 2011) in the cell pairs reported here (Figures 3, 4, and 5). This columnar processing trend, with the same tunning bias of L2/3 and L5 cells, reported in 81% of the cell pairs in *Spatial* trials was also present in the same percentage during *Object* trials, although the direction of tuning biases in the same minicolumn varied between these two trial contingencies.

Figures 5B and C show a very important distinction with respect to PFC inter-laminar processing which illucidates markedly why animals were less efficient in performing *Spatial* vs. *Object* types of trials with the same delays (Figure 5D) in the same behavioral sessions. The reduction in L2/3-L5 cell pair correlation on *Spatial* trials shown in Figure 5C, reflects a difference related to a state controlled by “prior” trial specific instruction (Figure 1A Focus signal) and suggests a lack of contextual encoding sufficient to maintain the same level of interlaminar communication. This is supported also by the demonstration of the independent influence of trial delay shown in Figure 4 which clearly had a greater influence on *Spatial* vs. *Object* trials. In addition, the electrochemical measurement of glutamate concentration in Layer 2/3 (Figures 5E–F) suggests that different networks, circuits, or even possibly, interlaminar columns of PFC neurons, differentially support the processing of *Spatial* vs. *Object* trials. Thus, Inter-laminar processing likely underlies the putative “executive function” of this brain region. These unique neural recordings demonstrate that relations between prefrontal neurons that encode and process information between cortical layers via minicolumns are likely relevant factors involved in executive dysfunction in which inter-laminar disruption could be the basis for the cognitive impairment as shown recently (Hampson et al., 2011, 2012; Opris et al., 2012). This was verified by the fact that delivery of the appropriate firing pattern with MIMO model derived electrical stimulation in the same L5 neural firing pattern as during successful execution of the MR in the task, improved performance when more distracter images were present (Figure 7D). However the fact that MIMO stimulation also facilitated performance by avoiding a different type of error with respect to retaining and implementing the “rule” for the type of trial (*Object* or *Spatial*) being executed (Figure 7E), suggests that closing PFC columnar loops activates a process that normally functions to enhance cognitive decision making in NHPs performing tasks that require retention of the contexts in which target selections are made.

### Conflict of interest statement

The authors declare that the research was conducted in the absence of any commercial or financial relationships that could be construed as a potential conflict of interest.
